# Detection of extended-spectrum β-lactamases in *Klebsiella pneumoniae*: comparison of phenotypic characterization methods

**DOI:** 10.12669/pjms.293.3576

**Published:** 2013

**Authors:** Hasan Ejaz, Ikram ul-Haq, Saqib Mahmood, Aizza Zafar, Muhammad Mohsin Javed

**Affiliations:** 1 Hasan Ejaz, M.Phil, Institute of Industrial Biotechnology, GC University, Lahore, Pakistan.; 2 Ikram-ul-Haq, PhD, Institute of Industrial Biotechnology, GC University, Lahore, Pakistan.; 3 Saqib Mahmood, PhD, Department of Human Genetics and Molecular Biology, University of Health Sciences, Lahore, Pakistan.; 4 Aizza Zafar, M.Phil, Department of Microbiology, The Children’s Hospital & Institute of Child Health, Lahore, Pakistan.; 5 Muhammad Mohsin Javed, PhD, Institute of Industrial Biotechnology, GC University, Lahore, Pakistan.

**Keywords:** K. *pneumoniae*, ESBL, DDST, CLSI, Comparison of detection methods

## Abstract

***Objective:*** Extended-spectrum β-lactamase producing *K. pneumoniae *is a serious threat to the patients. This manuscript shows the comparison of phenotypic characterization methods used for ESBL *K. pneumoniae *and frequency distribution of these isolates in various clinical samples.

***Methodology:*** Eleven different types of pathological samples collected on various time intervals were analyzed. *K. pneumoniae* were identified with API 20E system (bioMerieux) and initial screening of ESBL *K. pneumoniae* was performed using the ceftazidime antimicrobial disc. Double-disc synergy test (DDST) and CLSI confirmatory test were compared for the phenotypic detection of ESBL *K. pneumoniae*.

***Results:*** A total number of 214 ESBL producing *K. pneumoniae* were isolated from various clinical samples. Frequency distribution of ESBL producing *K. pneumoniae* was found to be highest among blood 117 (54.7%) and urine 46 (21.5%) samples. Data regarding the use of various interventions among these patients showed most common presence of intravenous line 209 (97.7%) and urinary catheters 46 (21.5%). Comparison of DDST and CLSI confirmatory test showed that the DDST detected 145 (67.8%) isolates while 213 (99.5%) ESBL *K. pneumoniae *were characterized by CLSI confirmatory test.

***Conclusion:*** The use of CLSI confirmatory test is very efficient in the early detection of ESBL *K. pneumoniae* especially when the facilities for molecular characterization are not available.

## INTRODUCTION

Extended-spectrum β-lactamases (ESBLs) are the enzymes, mostly encoded by plasmids in result of mutation due to which bacteria show resistance to various β-lactam antibiotics including cephalosporins and monbactams.^1^ Beyond one hundred and fifty various ESBLs have been described and majority of them belong to class A enzymes (SHV, TEM and CTX-M).^2^ ESBLs are most commonly found in bacteria (G^-^) especially the members of *Enterobacteriaceae *family*. *An increase in prevalence of ESBLs has been documented in recent years but it varies in different geographical areas of the world.^3^

*Klebsiella pneumoniae *is the most important and most common infectious pathogen in the hospitals environment and is mainly responsible for pneumonia, urinary tract infections, neonatal septicemia and wound infections among children.^4^ Apart from the other members of *Enterobacteriaceae *family, ESBLs are highly prevalent in *K. pneumoniae.*^5^ Global data show that the rate of ESBL producing bacteria was highest among *K. pneumoniae *isolated from fourty four percent in America followed by twenty two percent Asia pacific, thirty three percent in Europe and seven percent in North America.^6^ Many nosocomial outbreaks caused by ESBL *K. pneumoniae *have been reported among paediatric patients. ESBL *K. pneumoniae *are associated with high mortality rate among children.^7^

Many tests have been recommended for the detection of ESBL production in vitro*. *The most commonly used methods include double disc synergy test, combined disc method and E-test. Several automated systems have also been developed for detection and some laboratories use molecular methods for detection of ESBL phenomenon.^8^ There are limited number of antibiotic options available to treat the infections caused by ESBL producing strains.^9^ Present study was conducted with the objective, to evaluate the frequency of ESBL producing *K. pneumoniae *and comparison of detection methods among various clinical samples collected from a tertiary care children hospital. The early detection of ESBL producing *K. pneumoniae *strains can help to reduce high morbidity and mortality in paediatric patients.

## METHODOLOGY

The study was conducted at the Microbiology Department of The Children’s Hospital and Institute of Child Health Lahore, Pakistan, from May 2010 to February 2012. Eleven pathological samples including blood, cerebro-spinal fluid (CSF), urine, sputum, peritoneal dialysis catheters, tracheal secretions and pus were collected from paediatric patients were analysed during the study period. The patient’s history regarding the use of various interventions like intravenous line, urinary catheters, endotracheal tube, lumber puncture, peritoneal dialysis catheters, exchange transfusion, nasogastric tube, surgery, central venous pressure line and tracheostomy was also noted. The Brain Heart Infusion Broth (BHI) was used to inoculate the blood samples and after a period of incubation they were sub-cultured on solid media. All of the clinical samples were cultured on different solid media of Blood, Chocolate and MacConkey agar plates (90mm) while the urine samples were cultured only on Cystine Lysine Electrolyte Deficient Medium (CLED). An overnight incubation of these culture plates was done at 37±1ºC in an incubator. The bacterial strains were identified using API 20E system (bioMerieux)*.* Only the *K. pneumoniae* strains were included and processed further for ESBL detection. The culture media and antibiotic discs were purchased from Oxoid.

The *K. pneumoniae* strains were screened as ESBL screening-positive on the basis of resistance to ceftazidime. The screen positive *K. pneumoniae *strains were further processed for double disc synergy test (DDST) and Clinical Laboratory Standard Institute (CLSI) confirmatory test. In DDST, a disc of amoxicillin-clavulanic acid (AMC) was placed in the center of Mueller-Hinton agar plate (90mm) at 20mm distance to ceftazidime (CAZ 30μg) and cefotaxime (CTX 30μg). ESBL production was detected by the appearance of key hole effect due to the enhanced activity of ceftazidime and cefotaxime with clavulanic acid.

Both DDST positive and negative *K. pneumoniae* strains were analysed by CLSI confirmatory test. The CLSI confirmatory test was performed with of ceftazidime (CAZ 30μg) and cefotaxime (CTX 30μg) alone and using a combined disc of ceftazidime-clavulanic acid and cefotaxime-clavulanic acid (CAZ/CLA and CTX/CLA 30/10 μg). The CLSI confirmatory test was considered positive when the inhibition zone produced by the combined effect of ceftazidime or cefotaxime and clavulanic acid increased ≥5 mm than ceftazidime or cefotaxime without the clavulanic acid.^10^

## RESULTS

A total number of 710 *K. pneumoniae* were isolated during the study period. Out of 710 positive culture 214 (30.1%) were ESBL screening-positive* K. pneumoniae *and 496 (69.9%) were screening-negative positive* K. pneumoniae*. 

Initial screening of *K. pneumoniae* isolates with ceftazidime showed positivity in all of the isolates. Comparison between DDST and CLSI confirmatory test showed that 145 (67.8%) isolates were identified by DDST and 213 (99.5%) by using CLSI confirmatory test (Table-I). The CLSI confirmatory test had significantly (p<0.0001) higher sensitivity. Frequency distribution of ESBL *K. pneumoniae* was found to be highest in the blood samples 117 (54.7%) followed by urine 46 (21.5%), endotracheal tube 13 (6.1%), cerebro-spinal fluid 13 (6.1%) and peritoneal dialysis catheters 10 (4.7%). The rest of the samples showed the lesser occurrence of ESBL producing *K. pneumoniae* (Table-II).

The frequency of various interventions among the 214 ESBL *K. pneumoniae* positive cases showed that most of the patients had intravenous line 209 (97.7%) followed by urinary catheters 46 (21.5%), endotracheal tube 18 (8.4%), lumber puncture 18 (8.4%) and peritoneal dialysis catheters 16 (7.5%). The less common interventions were exchange transfusion, nasogastric tube, surgery, central venous pressure line and tracheostomy (Table-III).

**Table-I:** Comparison of two ESBL detection methods (n= 214).

*Test*	*No. of positive isolates*	*(%)*
Double disc synergy test*	145	67.8
CLSI confirmatory test* (combined disc)	213	99.5

* The accuracy of the DDST test is significantly lower than that of CLSI test (p<0.0001, McNewmar’s test).

**Table-II:** Frequency distribution of ESBL producing *K. pneumoniae* from various clinical samples (n=214

*Specimens *	*ESBL producing * *K. pneumoniae*	
	*No.*	*%*
Blood	117	54.7
Urine	46	21.5
Endotracheal tubes	13	6.1
Cerebro-spinal fluids	13	6.1
Peritoneal dialysis catheters	10	4.7
Tracheal secretions	4	1.9
Pus	3	1.4
Central venous pressure lines	2	0.9
Wound swabs	2	0.9
Ear swabs	2	0.9
Pleural fluids	2	0.9

**Table-III:** Frequency of various interventions among the ESBL positive patients (n=214).

*Intervention*	*No.*	*%*
Intravenous line	209	97.7
Urinary catheter	46	21.5
Endotracheal tube	18	8.4
Lumber puncture	18	8.4
Peritoneal dialysis catheter	16	7.5
Exchange transfusion	6	2.8
Nasogastric tube	5	2.3
Surgery	4	1.9
Central venous pressure line	2	0.9
Tracheostomy	1	0.5

## DISCUSSION

ESBL producing *K. pneumoniae* is an important clinical pathogen responsible for life threatening infections especially among neonates and infants. This study provides the recent data about the frequency distribution and comparison of phenotypic detection methods used to characterise ESBL producing *K. pneumoniae* from various clinical samples of children.

In our study frequency of ESBL producing *K. pneumoniae* is 30.1% which is lower than few other studies. A research work conducted in Pakistan Institute of Medical Sciences reported a high frequency of 70% ESBL producing K. pneumoniae.^11^ Another research study conducted in different hospitals of Iran reported 59.2% ESBL *K. pneumoniae*.^12^ A study conducted in Lucknow, India reported 56% ESBL *K. pneumoniae* among neonates who had septicemia.^13^ Frequency of ESBL producing *K. pneumoniae* is not very high in our study in comparison with other studies. The prevalence of ESBL *K. pneumoniae* varies from one institution to another depending upon the level of cleanliness and infection control measures.

Comparison of DDST and CLSI showed that most of the ESBL positive *K. pneumoniae* were detected by CLSI (99.5%) confirmatory test (p<0.0001) than DDST (67.8%). CLSI even detected ESBLs in those 69 isolates which were missed by DDST. Dalela worked on ESBLs at Jhalawar Medical College in India, used DDST and CLSI for detection of ESBLs in gram-negative bacteria. Out of his 135 screening positive ESBL isolates, DDST detected ESBLs in 122 (90%) isolates while CLSI detected ESBLs in all isolates (100%).^14^ Similarly another study conducted at Microbiology department of Sher-i-Kashmir Institute of Medical Sciences, Kashmir-India also compared DDST and CLSI methods in detection of ESBL production in *K. pneumoniae*. Out of 92 screen positive isolates, DDST detected ESBL production in 32 (34.8%) isolates while CLSI detected ESBLs in 72 (78.3%) isolates.^3^ A study conducted by Dhara et al reported that DDST detected only 75% isolates with ESBLs while CLSI detected ESBLs in 85.4% of *K. pneumoniae* isolated from neonatal intensive care unit patients.^15^ In our experience CLSI missed only 1 (0.5%) isolate. One group of researchers reported 15.2% false negativity with CLSI methods among the screening positive isolates.^16^ Our findings supports the results of most the previous findings suggesting that CLSI confirmatory test is more reliable in detection of the ESBL *K. pneumoniae* than the other phenotypic methods.

Frequency distribution of ESBL producing *K. pneumoniae *varied among different pathological samples, a high percentage of 54.7% was isolated from blood followed by urine (21.5%), cerebro-spinal fluid (6.1%) and pus (1.4%). Frequency of ESBL producing *K. pneumoniae *in different clinical samples was also reported in a study conducted at 1000 bed teaching hospital, Spain. A very high percentage of *K. pneumoniae* was found in blood (40%) and catheters (36%) followed by respiratory fluid (26%), urine (23%) and pus (18%).^17^ Similarly a research work conducted at Agha Khan University, Karachi reported high percentage of ESBL positive *K. pneumoniae *in blood (60%), urine (24%) and wound (6%).^18^ Ahmad et al also reported more of the ESBL producing *K. pneumoniae *strains from blood (44.9%) while less frequency was observed in urine (16.2%), tracheal secretions (10.9%), wound (5.9%) and endo-tracheal tip (5.9%).^3^ The results of these studies are in agreement with our findings indicating the detection of most of the ESBLs in patients with bacteremia.

In our study we found that most of the patients with ESBL *K. pneumoniae* infections had some interventions like intravenous lines (97.7%) and urinary catheters (21.5%). A study reported the association of invasive procedures like urinary catheters 6 (26.1%), surgeries (26.1%) and mechanical ventilation 3 (13.0%) among the ESBL positive cases of *K. pneumoniae*.^19^ In another study the most common interventions found in 79 cases of ESBL *K. pneumoniae *infections were central venous catheters 72 (91.1%), urinary catheters 70 (88.6%), surgeries 66 (83.5%), nasogastric tube 56 (70.9%) and tracheostomy 37 (46.8%).^20^ The ESBL *K. pneumoniae* infections can vary in patients depending upon the various interventions and how they were used. The intravenous lines and catheters can be a good source infection if not used aseptically.

## CONCLUSION

ESBL producing *K. pneumoniae *is a serious concern for the hospitals. It is associated with high morbidity among paediatric patients which results in high cost of treatment. An appropriate antimicrobial therapy can only be started timely with the early detection of ESBLs. The CLSI confirmatory test can be used reliably especially when the facilities for molecular charaterization are not available. The detection of ESBL producing strains will also help to establish and implement a strict infection control policy to stop the spread of ESBL *K. pneumoniae*.

## Authors Contribution

*Hasan Ejaz:* The idea of topic, experimental work and wrote the manuscript.

*Ikram-ul-Haq:* Supervision of experimental work and writing of the manuscript.

*Saqib Mahmood: *Co-supervision in experimental work and interpretation of results.

*Aizza Zafar:* Provided the facilities and technical support for research.

*Muhammad Mohsin Javed: *Helped in manuscript drafting and data analysis.
